# The Neural Basis of Object-Context Relationships on Aesthetic Judgment

**DOI:** 10.1371/journal.pone.0003754

**Published:** 2008-11-19

**Authors:** Ulrich Kirk

**Affiliations:** Wellcome Laboratory of Neurobiology, Anatomy Department, and Wellcome Department of Imaging Neuroscience, University College London, London, United Kingdom; James Cook University, Australia

## Abstract

The relationship between contextual information and object perception has received considerable attention in neuroimaging studies. In the work reported here, we used functional magnetic resonance imaging (fMRI) to investigate the relationship between aesthetic judgment and images of objects in their normal contextual setting versus images of objects in abnormal contextual settings and the underlying brain activity. When object-context relationships are violated changes in visual perception and aesthetic judgment emerges that exposes the contribution of vision to interpretations shaped by previous experience. We found that effects of context on aesthetic judgment modulates different memory sub-systems, while aesthetic judgment regardless of context recruit medial and lateral aspects of the orbitofrontal cortex, consistent with previous findings. Visual cortical areas traditionally associated with the processing of visual features are recruited in normal contexts, irrespective of aesthetic ratings, while prefrontal areas are significantly more engaged when objects are viewed in unaccustomed settings.

## Introduction

Objects are usually associated with the context in which they appear, which provide expectations about which objects are likely to appear in a given contextual scene. The importance of prior knowledge in determining how contextual association influences object recognition has been well-documented in behavioural studies. These studies show that objects appearing in familiar or consistent contextual scenes are detected more accurately and processed more quickly than objects appearing in unfamiliar or inconsistent contexts [1]–[7]. This suggests that objects and the context in which they appear are processed interactively, thus facilitating the perceptual processes involved in visual object recognition [8], [9]. Such behavioural findings have recently been adapted to neuroimaging studies [10]–[14]. Bar and colleagues (2003) investigated the cortical correlates of contextual associations by comparing objects with strong contextual associations presented in their normal surroundings (e.g. a blender) with objects with weak contextual associations (e.g. a mobile-phone). They found that the former elicited greater activity than the latter in the parahippocampal gyrus and in the retrosplenial cortex, which together comprise a cortical network processing contextual associations during object recognition [8], [15].

In extending this work we wanted to investigate the neural effects of context (normal and abnormal) on aesthetic judgment. Such an approach promised us insights in the general field of neuroaesthetics. Indeed the stimuli that we prepared for this study were inspired by the work of the Surrealist artist René Magritte (1898–1967) who, in his compositions, gave visual objects primacy by divorcing them from the surroundings with which they are usually associated. Likewise, stimulus novelty has been shown to have a significant effect on aesthetic ratings [16], [17]. Because the effect of perceptual novelty is so explicit in the work of Magritte, the use of stimuli derived from this approach allowed us to analyse the influence of novelty on aesthetic ratings.

**Figure 1:**
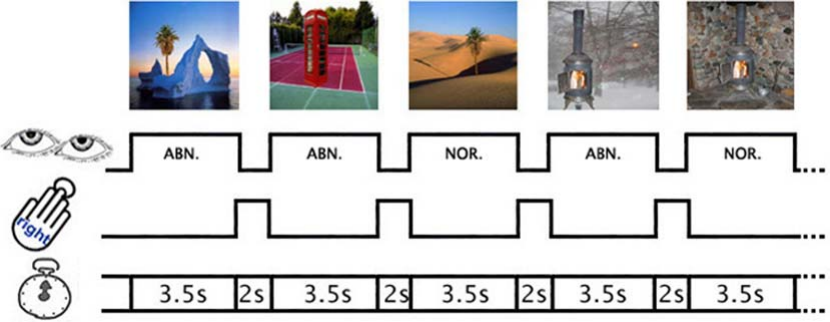
Experimental paradigm. Examples of the stimulus material used in the fMRI experiment are shown; the stimulus material was constructed in pairs such that objects were presented in both a normal and an abnormal contextual setting. Stimulus presentation was 3500 ms followed by a response-period (black screen) where subjects were required to indicate their aesthetic rating (appealing, neutral, unappealing) via button press (2000 ms).

In the present study, the critical components of interest were objects that were either consistent or inconsistent with their normally associated context (fig. 1), in an event-related fMRI approach. As object-context relationships are learnt over time, based on real life pairing frequencies [18], conflicting or abnormal trials can be experimentally regulated by altering the object-context pairings into less probable ones. Thus, by varying the setting in which objects appeared, we were able to assess the neural response to the relationships between the two relative to the aesthetic ratings attributed to each stimulus. Previous work has shown that perceived beauty correlates with activity in the medial aspects of the orbito-frontal cortex (OFC), with the intensity of activity there being related to the declared experience of the beauty of the viewed work of art [19]. We wanted to supplement this work by examining the relationship between the declared experience of the aesthetic satisfaction derived from viewing the images we presented, whether in normal or abnormal contexts, and activity in the relevant cortical areas. To accommodate this experimental aim we applied linear parametric contrasts and interactions to investigate effects of normal and abnormal context on aesthetic ratings.

Additionally, the experimental design allowed us to test whether the neural pathways engaged when we view objects in normal settings are significantly different from those engaged when we view them in abnormal ones, irrespective of aesthetic ratings. The chances that this would be so seemed high, given that departures from expectation in general results in activation of areas that are not found to be active when the percept is consistent with prior experience. In particular, many studies have shown that the dorso-lateral prefrontal cortex (DLPFC) and especially Brodmann area 46 is active when subjects experience stimuli, whether visual or otherwise, that depart from expectation. Among such stimuli are objects dressed in un-natural colours [20], oddball tasks [21], infrequent events [22]–[24], general perceptual and emotional deviance [25] and irregular temporal patterns [26]. Our general hypothesis was, therefore, that when objects are viewed in unusual or unnatural settings, the activity will always involve the DLPFC. Furthermore, as subjects were instructed to rate each stimulus according to aesthetics it encourages subjects, although implicitly, to ignore contextual information. This experimental design extends previous studies that used behavioural tasks where subjects were required to attribute explicit attention to contextual information in the stimuli-material [11], [14]. Specifically, we were able to investigate whether the cortical context network [9], such as the parahippocampal gyrus and the retrosplenial cortex, would be influenced by this change.

## Materials and Methods

### Subjects

Fifteen right-handed subjects (six females) with a mean age of 24.4 years participated in the study. All gave informed consent in accordance with the Declaration of Helsinki, and the Ethics Committee of the National Hospital for Neurology and Neurosurgery, London, UK, granted ethics approval for the study. All had normal or corrected-to-normal vision, and none had a history of neurological or psychiatric disorders. Subjects were all undergraduate or graduate students.

### Stimuli and task

Visual chromatic stimuli belonging to two categories, normal and abnormal photographs, 120 in total, were selected from online sources. Abnormal versions were manipulated in Photoshop (version 7.0, Adobe, USA) by superimposing a photo of an object onto a different contextual background such that the object was in the foreground and appeared against a background with which the object is not usually associated (abnormal conditions) (fig. 1). Any image noticeably distorted (e.g. in proportion or illumination) by this process was excluded from the stimulus pool. The normal versions consisted of photos with an object against a background with which it is normally associated (normal conditions). The stimulus material was paired, such that the same objects were presented in normal and abnormal context. The experimental protocol consisted of an event-related design in which subjects were scanned while being presented with each of the stimuli only once. All trials were presented in a pseudorandom order, and trials were counterbalanced across subjects meaning that half of the subjects saw the abnormal version of a given stimuli before its normal counterpart, while this was reversed for the other half of subjects. A trial began with a fixation cross for 500 ms positioned at the centre of the screen against a black background. Each stimulus was presented at the centre of a computer screen on a black background for 3500 ms. This was followed by a response-period of 2000 ms (black screen) in which subjects were instructed to decide whether they found each stimulus aesthetically ‘appealing’, ‘unappealing’ or ‘neutral’. Subjects were instructed to passively view each image during the 3500 ms stimulus presentation. They could make their rating at any time during the response-period, by pressing one of three buttons on a response key-pad corresponding to the rating options with their right hand. Prior to scanning, subjects were instructed in the aesthetic rating task and were subsequently trained using demonstration stimuli, which were not included in the scanning session, until they were accustomed with the aesthetic rating task. It was imperative that the aesthetic rating task was presented in real-time (i.e. during scanning) in order to measure the oddball-effect on aesthetic ratings. The alternative would be to present the aesthetic rating task either pre- or post-scanning. However, this would inevitably have introduced a bias by compromising the oddball-effect (i.e. abnormal conditions). The stimuli were presented at a screen resolution of 1024×768 pixels displayed at a visual angle of 24×18°, and centred in a 500×500 pixel resolution surrounded by a black background. Stimuli were presented and responses collected using COGENT 2000 Graphics (http://www.vislab.ucl.ac.uk/Cogent/) running in Matlab (Mathworks Inc.). Total scanning-time per subject was 12 min. in one session. The stimuli were back-projected via a LCD projector onto a transparent screen positioned over the subjects' head and viewed through a tilted mirror fixed to the head coil.

### Data acquisition

The functional imaging was conducted by using a 1.5T Siemens Vision fMRI scanner (Siemens, Erlangen, Germany) to acquire gradient T2* weighted echo planar images (EPI) to maximize the blood oxygen level-dependent (BOLD) contrast (TE, 40 ms; TR, 3.42 s; flip angle, FA = 90°). Each functional image was acquired in a descending sequence comprising 38 axial slices each 2.5 mm thick, consisting of 64×64 voxels. This gave near whole-brain coverage, excluding the cerebellum. Each session consisted of 220 volumes with the first 5 volumes being discarded to allow for T1 equilibration effects. After every functional scan, a T1 weighted structural sequence was acquired, using a phased array head coil to provide high-resolution anatomical detail. The structural image was co-registered to the EPI images, thus allowing functional data to be overlaid on a high-resolution anatomical image.

### Data analysis

Image pre-processing and data analysis was performed using SPM2 (Wellcome Department of Imaging Neuroscience, London, UK). The EPI images were realigned spatially [27]. This was followed by temporal realignment, which corrected for slice-time differences using the middle slice as reference. Images were then normalized to the Montreal Neurological Institute (MNI) template provided in SPM2. Finally a spatial filtering was performed by applying a Gaussian smoothing kernel of 12 mm full width at half-maximum.

**Figure 2:**
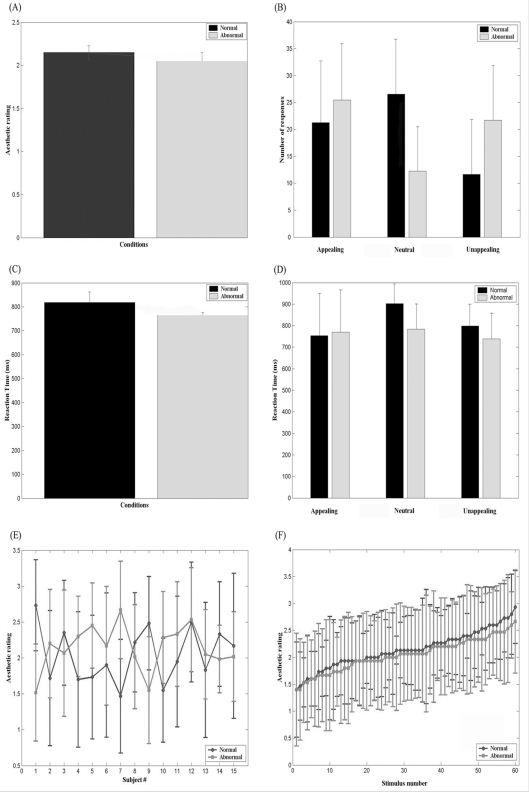
Behavioural data (n = 15) collected in the fMRI study. (A) Mean aesthetic ratings for the two stimulus conditions, normal and abnormal, across subjects. (B) Distribution of aesthetic responses for the two stimulus conditions, normal and normal, across the three rating bins (appealing, neutral, and unappealing). (C) Mean reaction times (RT) for each of the two stimulus conditions. (D) Distribution of RTs for the two stimulus conditions across the three rating bins. (E) Mean aesthetic ratings for normal and abnormal conditions for each subject, compiled across stimuli. (F) Stimuli are shown distributed across the range of ratings. Stimuli are rank-ordered by mean aesthetic ratings separate for each condition, compiled across subjects. Error bars are standard deviation is all displays (A–F).

Following pre-processing a general linear model was applied to the fMRI time-series where stimulus onset was modeled as single impulse response function and convolved with SPM2's canonical haemodynamic response function. Data from 15 subjects were applied in a random-effects (RFX) multiple regression analysis, whereby the reliability of the measurements was assessed in relation to the between subject variance [28]. We modelled six regressors of interest: abnormal-appealing, abnormal-neutral, abnormal-unappealing, normal-appealing, normal-neutral and normal-unappealing. Button–press and question-duration times were modelled as regressors of no interest. Residual effects of head motion were corrected for by including the six estimated motion parameters for each subject as regressors of no interest. For the analysis a high pass filter with a cut-off frequency at 1/128 Hz was applied. As the behavioural data demonstrated a statistically significant interaction between stimulus category and rating category (fig. 2B), further regressors were applied to separate the data in order to control for these behavioural differences between conditions. For each subject the frequency of each of the three rating bins was balanced, such that there were no differences in ratings and reaction-times between normal and abnormal conditions as such a difference would confound the results with context-effects or aesthetic ratings or a combination of the two. The residual rating bins from this process were for each subject put in separate regressors.

The resultant parameter estimates for each regressor (at each voxel) were compared using t-tests, allowing us to test for correlations of the fMRI BOLD signal and the parameters of interest. The statistical results given are based on a single-voxel t-statistics corresponding to p<0.001, uncorrected for multiple comparisons. In order to correct for multiple comparisons in interpreting these results, small volume corrections (SVC) [29] with a sphere of 10 mm radius were used for medial OFC of which we had prior anatomical hypothesis. The SVC was performed using the co-ordinates provided by a previous study for medial OFC [19]. Before using SVC, we transformed coordinates given by this previous study from Talairach space to MNI space (www.mrc-cbu.cam.ac.uk). The co-ordinates of all activations are reported in MNI space.

## Results

### Behavioural results

Examination of the behavioural data collected during scanning showed that abnormal conditions were rated as being on average 2.05 (std = 0.1) and normal conditions were on average 2.15 (std = 0.08) on the aesthetic rating scale (1 = unappealing; 2 = neutral; 3 = appealing). Statistical analysis showed no significant difference between the two conditions (paired t = 0.88; df = 14; p>0.39) (fig. 2A). Subject-averaged reaction times (RT) were 764 ms (std = 13.6) for abnormal conditions and 818 ms (std = 44.4) for normal ones. The two conditions did not produce significant differences in RT (paired t = 1.37; df = 14; p>0.3) (fig. 2C). Thus, an analysis of the average ratings and RT results shows that subjects did not respond significantly different to abnormal and normal conditions.

We next inspected whether the frequency of each rating category differed across stimulus category (fig. 2B). A stimulus category (abnormal, normal)×rating category (appealing, neutral, unappealing) ANOVA revealed no significant main effect for stimulus category (ANOVA, F(1,1) = 0, p>0.99) or rating category (ANOVA, F(1,2) = 2.59, p>0.08). There was a significant interaction between stimulus category and rating category (ANOVA, F(1,2) = 9.15, p<0.0003).

Although subjects rated the normal and abnormal images as being on average equally appealing, they were more likely to attribute more extreme ratings to abnormal images (i.e. either appealing or unappealing) whereas normal images were more likely to evoke aesthetic ratings that were neither appealing nor unappealing, which is reflected in the interaction between stimulus and rating category. This trend in the data yielded an inter-subject response pattern, as we found no significant differences between subjects and rating category (ANOVA, F(1,14) = 1.26, p>0.27). This confirms an inter-subject response pattern displaying a tendency to attribute a binary rating strategy in abnormal conditions (i.e. appealing or unappealing responses) compared to normal conditions. Thus, this confirms that the binary rating strategy was not driven by a subset of subjects rating abnormal images with a higher frequency of appeal, while other subjects attribute a higher frequency of unappealing responses. Furthermore, the mean rating of normal and abnormal conditions for each subject show that subjects were fairly consistent with each other, although particular two subjects displayed extreme preference for either normal (subject#1) or abnormal (subject#7) conditions (fig. 2E). Despite the large standard deviations the stimuli displayed inter-subject consistency and individual stimuli pairs were not influenced significantly by normal or abnormal conditions (fig. 2F). Taken together, these results show a general response pattern across subjects to attribute a binary rating strategy suggesting that novelty/abnormality influence aesthetic ratings [16], [17].

### fRMI results

**Figure 3:**
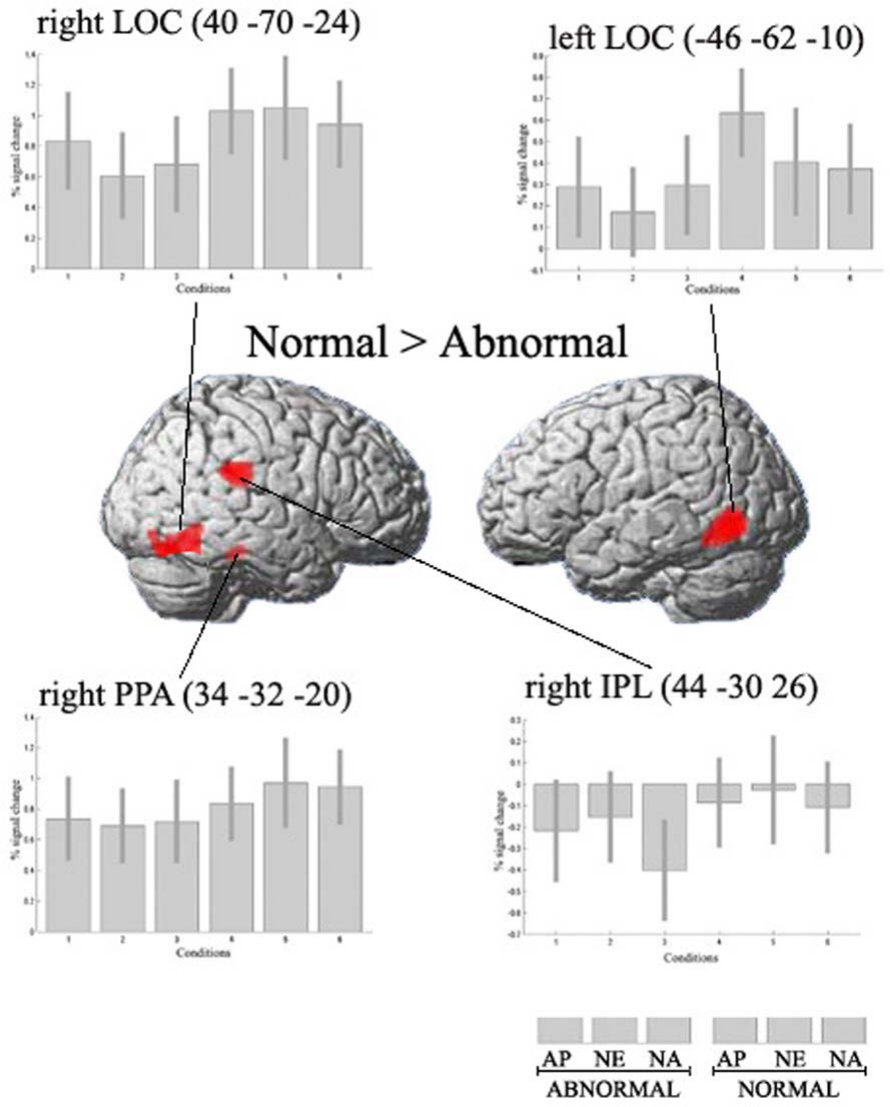
Main effect [normal>abnormal] revealed significant activity (p<0.001, uncorrected) in bilateral LOC, bilateral parahippocampal gyrus (PPA), and right inferior parietal lobule (IPL). Specific coordinates of activations are given in Table 1. Activations are surface rendered on the canonical SPM structural image. Bar plots show differences in parameter estimates between normal and abnormal conditions. AP = appealing; NE = neutral; NA = unappealing. Error bars indicate 90% confidence interval.

**Figure 4:**
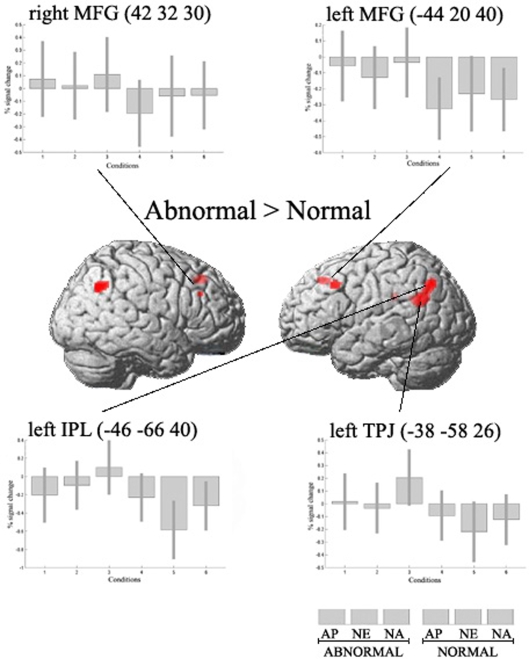
Main effect [abnormal>normal]. Significant voxels (p<0.001, uncorrected) were bilateral middle frontal gyrus (MFG), anterior cingulate (not shown), left temporo-parietal junction (TPJ), bilateral inferior parietal lobule (IPL) and retrosplenial cortex (not shown). Specific coordinates of activations are given in Table 1. Activations are surface rendered on the canonical SPM structural image. Bar plots show differences in parameter estimates between abnormal and normal conditions. AP = appealing; NE = neutral; NA = unappealing. Error bars indicate 90% confidence interval.

We studied two main effects: [normal conditions>abnormal conditions] and [abnormal conditions>normal conditions]. In the first contrast we observed significant activations in the lateral occipital complex (LOC) bilaterally (fig. 3). We also found activation in the parahippocampal gyri bilaterally, and in the right inferior parietal lobule (Table 1). In the inverse main effect [abnormal conditions>normal conditions], significant activations were seen in the middle frontal gyrus bilaterally (MFG) and in the anterior cingulate gyrus, overlapping with the medial superior frontal gyrus (fig. 4). We also found activity in left temporo-parietal junction (TPJ), inferior parietal lobule (IPL) bilaterally and left retrosplenial cortex (Table 1).

**Table 1:** Location of brain regions that respond to main effects of context.

Brain region	Peak MNI coordinates			z score	Number of voxels
[Normal>Abnormal]					
L. lateral occipital cortex (LOC)	−46	−62	−10	3.81	266
R. lateral occipital cortex (LOC)	40	−70	−24	2.94	126
R. inferior parietal lobule (IPL)	44	−30	26	3.73	233
R. parahippocampal gyrus (PPA)	34	−32	−20	2.94	40
L. Parahippocampal gyrus (PPA)	−26	−36	−14	2.64	8
[Abnormal>Normal]					
L. temporo-parietal junction (TPJ)	−38	−58	26	4.39	200
L. inferior parietal lobule (IPL)/angular gyrus	−46	−66	40	3.50	109
R. inferior parietal lobule (IPL)/angular gyrus	58	−60	38	3.65	91
L. middle frontal gyrus (MFG)	−44	20	40	3.50	52
R. middle frontal gyrus (MFG)	42	32	30	3.19	6
L. superior frontal gyrus/anterior cingulate	−4	30	44	3.50	95
L. retrosplenial cortex	−6	−34	28	3.45	15
	−14	−50	26	3.30	22

Activations are shown at (p<0.001, uncorrected). L. left hemisphere; R, right hemisphere.

**Figure 5:**
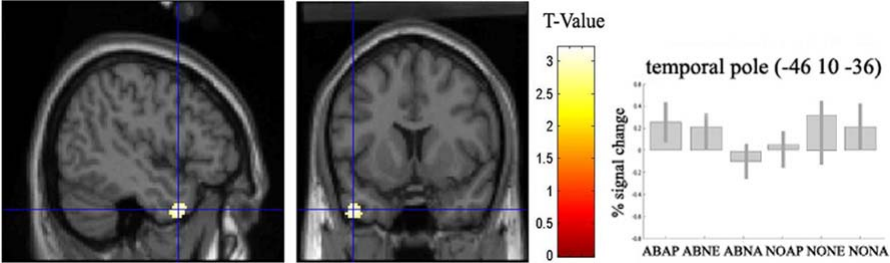
Interaction effect [abnormal appealing>abnormal unappealing]>[normal appealing>normal unappealing]. Activation in left temporal pole (−46, 10, −36; k = 115, z = 3.11) significant at (p<0.001, uncorrected) is displayed on saggital and coronal sections of the canonical SPM structural image. On the right are displayed parameter estimates for voxels in left temporal pole. Bar plots show differences in parameter estimates between abnormal and normal conditions. AP = appealing; NE = neutral; NA = unappealing. Error bars indicate 90% confidence interval.

We next inspected interaction effects of context on aesthetic ratings to assess different brain areas involved in aesthetic judgment for normal and abnormal context. This analysis was motivated by the significant interaction between stimulus category and rating category observed in the behavioural data (see fig. 2B). Thus we performed the interaction [abnormal appealing>abnormal unappealing]>[normal appealing>normal unappealing]. The left temporal pole showed distinct specificity to a modulation of aesthetic ratings for abnormal conditions vs. normal conditions (fig. 5). The inverse interaction, revealed no suprathreshold activity (p<0.001, uncorrected; not shown).

To further determine the effect of context on aesthetic ratings, linear parametric contrasts were used. We looked for voxels at which the BOLD signal showed a positive and a negative linear correlation with aesthetic ratings separate for normal and abnormal conditions.

**Figure 6:**
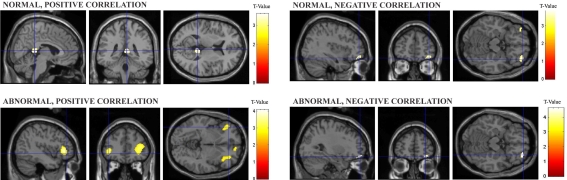
Differential linear correlation patterns with aesthetic ratings for normal (upper panels) and abnormal conditions (lower panels). Upper left panel: Activity in right posterior cingulate (6 −40 10; k = 139; z = 3.44) was positively correlated with aesthetic ratings in normal conditions. Upper right panel: Negative correlations with aesthetic ratings for normal conditions activated right lateral OFC (34 48 −14; k = 94; z = 3.59) and left lateral OFC (−34 46 −14; k = 48; z = 3.38). Lower left panel: Positive linear correlations with aesthetic ratings for abnormal conditions resulted in activity in left inferior frontal gyrus (−38 34 4; k = 347; z = 3.89), right inferior frontal gyrus (38 28 −4; k = 324; z = 3.25) and right frontomedian cortex/frontal pole (12 54 10; k = 124; z = 3.0). Lower right panel: Negative linear correlations with aesthetic ratings for abnormal conditions recruited right lateral orbitofrontal cortex (36 50 −14; k = 88; z = 3.54). All activation were found at p<0.001, uncorrected. Activations are overlaid on a saggital, coronal and axial sections of the canonical SPM structural image.

For normal conditions, activity in the right posterior cingulate was positively correlated with aesthetic ratings, whilst bilateral lateral OFC showed a negative correlation (fig. 6). For abnormal conditions activity in right fronto-median cortex/frontal pole and bilateral inferior frontal gyrus was positively correlated with aesthetic ratings. Finally, right lateral OFC showed a negative correlation with aesthetic ratings for abnormal conditions (fig. 6).

**Figure 7:**
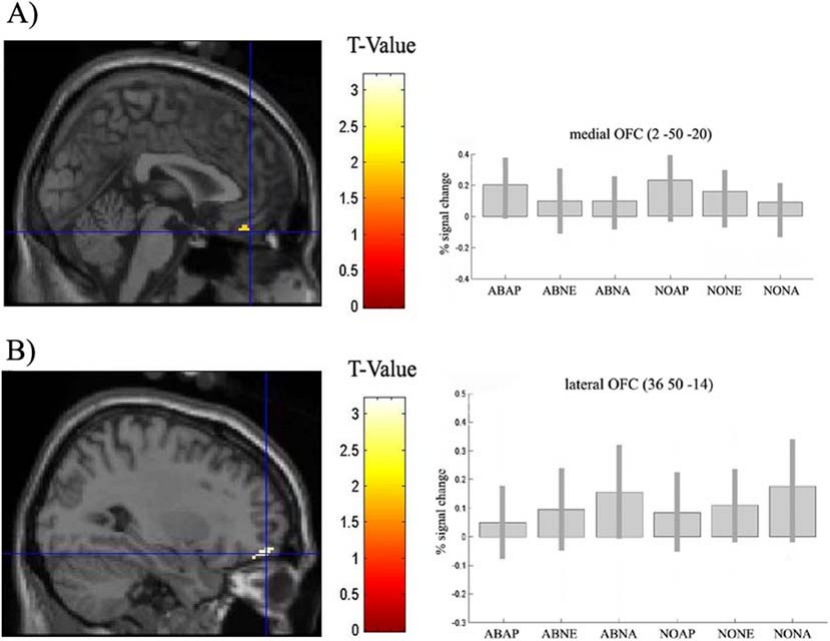
(A) A conjunction analysis (p<0.005^2^, uncorrected) was performed as a linear positive correlation with aesthetic ratings for normal and abnormal conditions. Voxels were identified in the medial OFC (2, 50, –20; k = 15; z = 2.79). Using SVC this region reached a significant level (p< 0.04, SVC). On the right are shown parameter estimates for voxels in medial OFC for both conditions for each rating bin. (B) Voxels at which activity showed a negative correlation with subjective aesthetic ratings irrespective of stimulus condition resulted in activity in right lateral OFC (36, 50, −14; k = 84; z = 3.52). One the right are shown parameter estimates for lateral OFC. Activations are displayed on saggital sections of the canonical SPM structural image. Bar plots show differences in parameter estimates between abnormal and normal conditions. AP = appealing; NE = neutral; NA = unappealing. Error bars indicate 90% confidence interval.

In order to identify areas involved in aesthetic evaluation irrespective of whether the viewed objects were in normal or abnormal contexts a conjunction analysis was applied. No regions were found to reach significance (p<0.001^2^, uncorrected). However when we dropped the level of significance (p<0.005^2^, uncorrected) we found that medial OFC showed a positive linear correlation with aesthetic ratings regardless of context (fig. 7A). This is consistent with previous studies which found that medial OFC correlates with beauty ratings in paintings [19], facial beauty [30], [31] and pleasantness of sound [32]. We therefore used small volume correction (SVC) constraining our analysis to this a priori region using the coordinates from a previous study [19]. Activity reached corrected significance in this area (p<0.04, SVC) (fig. 7A).

We next looked for voxels at which the BOLD signal showed a negative linear correlation with aesthetic ratings regardless of context. This conjunction analysis revealed activity in right lateral OFC (p<0.001^2^, uncorrected) (fig. 7B). The differential activation in the medial and lateral aspects of the OFC as identified in the conjunction analysis, support previous reports indicating that abstract reward and punishment representations occur in the medial and lateral OFC respectively [33] with coordinates overlapping those found in the present study.

## Discussion

The aim of this study was to investigate the relationship between aesthetic judgment and objects in their normal contextual setting versus objects in abnormal contextual settings. In the results reported here we found a medial-lateral trend in OFC suggesting a general response mechanism to aesthetic judgment regardless of context (normal and abnormal). In addition the results from the parametric analysis suggest a differential activation pattern that guide aesthetic judgment of normal and abnormal conditions. Specifically normal conditions activate areas associated with semantic memory. Whereas areas involved in monitoring discrepancies between expectation and experience in memory encoding are activated in aesthetic judgment of abnormal conditions. We furthermore observed differences in neural activity when objects are viewed in normal contexts compared to when they are viewed in abnormal ones irrespective of aesthetic ratings. Specifically, the LOC and parahippocampal areas were significantly more active when objects were presented in normal context while frontal and parietal areas became engaged when the relationship between object and context was unexpected.

### Contextual effects on aesthetic judgment

Although there were no significant effects of stimulus condition on average aesthetic ratings, there was a statistically significant tendency for subjects to use a binary rating strategy in abnormal conditions, i.e. rate stimuli as either appealing (20.7%) or unappealing (17%), whereas normal conditions received a significantly higher ratio of neutral response (22.5%). This response pattern was not significantly different across subjects, and may reflect a more careful evaluation before assigning an aesthetic value on novelty trials than normal trials [16], [17], which, although speculative, could indicate that subjects' inferred differential intentions to aesthetic judgments. Specifically, images of objects placed in an abnormal contextual settings lend themselves to aesthetic judgements (i.e. they are either appealing or unappealing), whereas objects placed within a normal contextual setting less obviously reflect aesthetic intention and are thus less likely to evoke aesthetic judgments (i.e. they are neither appealing nor unappealing). Such a differential employment of evaluation strategies would not necessarily be reflected in RTs in the present study, as subjects were instructed to view each stimulus passively (for 3500 ms) and to assign an aesthetic value to each image only after stimulus offset (see Material and Methods). Hence RTs might not have captured differential evaluation strategies. This view is substantiated by the failure to reach statistical significant differences in average RTs and in frequencies of each rating category for the two stimulus conditions. The interaction effect of contextual manipulation on aesthetic ratings resulted in a neural correlate in left temporal pole, a part of the brain thought to be implicated in semantic memory retrieval [34], [35]. However, the temporal poles also have a putative role in social and emotional processing, since they are frequently found to be active during complex emotional tasks such as viewing emotional picture sets, a response that is absent in simpler emotional tasks, such as emotional face or gaze perception tasks [36]. Several studies implicate the temporal pole in tasks that require subjects to analyse other peoples' emotions, intentions or beliefs [37]–[40], including humour comprehension [41]. Finally the temporal poles have been reported to be active in tasks that require explicit evaluation judgments such as emotional intensity [42], aesthetic judgments [43] and contextual framing that influence judgments of emotion in others [44]. Consistent with these findings, our data suggest that the left temporal pole may be engaged during evaluation of affectively salient information and thus a differential evaluation strategy in abnormal than in normal trials. A likely strategy employed by subjects in abnormal trials may be an attempt to organize novel object-context pairings into a framework of prior knowledge and use this information to guide and bias aesthetic judgments [43]. This may occur in a Baysian fashion by constantly updating and integrating information about the present context with previously stored knowledge [8], [15].

### Context modulates different memory sub-systems in aesthetic judgment

Contextual effects on aesthetic judgment were further investigated in the linear parametric analysis where two areas were positively correlated with aesthetic ratings for abnormal conditions, namely bilateral inferior frontal gyrus, corresponding to BA 45, and another region where the center of activation was located within the fronto-median cortex/frontal pole (BA 9/10). The latter region has been related to introspective evaluation of internal mental states, i.e. one's own thoughts and feelings, and tasks that require self-reference [38]. The former area has been implicated in oddball tasks [25]. Specifically, activation of the inferior frontal gyrus may be involved in the decoding of stimuli, whereas the fronto-median cortex may modulate the aesthetic evaluation of each stimulus. In contrast, activity in the posterior cingulate cortex was positively correlated with aesthetic ratings in normal conditions. In opposition to abnormal conditions, this activation signifies a behavioral bias to use semantic or episodic memory to guide aesthetic rating where subjects may have attributed ratings according to how familiar they were. Indeed, activity in the posterior cingulate has been related to successful memory retrieval [45], [46].

### Effects of aesthetic judgment irrespective of context

In the fMRI study of Kawabata & Zeki (2004), subjects viewed portrait, landscape, still life, and abstract paintings that they considered to be beautiful, compared to those that they considered neutral or ugly. Comparison of brain activity when viewing beautiful vs. ugly paintings yielded significant voxels in the medial OFC. The conjunction analysis in the present study revealed activation of medial OFC in a positive correlation with aesthetic ratings independent of stimulus conditions. The response of the medial OFC demonstrating sensitivity to the magnitude of aesthetic value is in accordance with studies on reward processing showing that the relative reward value of stimuli is reflected by the amplitude of neural activity in OFC [30]–[33]. The conjunction analysis further revealed activity in the right lateral OFC as a negative correlation with aesthetic ratings regardless of stimulus condition. This medial-lateral dissociation in the OFC has been observed in previous studies [33], [47]. According to these studies, medial OFC is related to monitoring the reward value, whereas lateral OFC activity is related to the evaluation of punishers that can lead to a change in behaviour. In these studies the right lateral OFC is invariably implicated, but rarely the left. The results of our activation are consistent with this theory.

### Contextual effects on object perception irrespective of aesthetic judgment

Objects viewed in abnormal contexts led to significantly more activity compared to normal contextual conditions in middle frontal gyrus bilaterally which forms part of the DLPFC, corresponding to BA 8, 9 and 46 and anterior cingulate cortex (ACC). This general cortical zone is activated whenever there is a departure from expectation. Examples include the viewing of objects dressed in colours not usually associated with them [20]; infrequent events [22]–[24], visual and auditory oddball tasks [21], general perceptual and emotional deviance [25] and irregular temporal patterns [26]. Previous functional imaging studies have implicated ACC in visual attention [48], emotional attention [49, and monitoring cognitive conflict [50]. Our observation of increased ACC activation in abnormal compared to normal conditions is consistent with these proposed cognitive roles for ACC. Similarly, the TPJ has been associated with a role in detecting novel or otherwise salient stimuli [22], [51], [52] in particular to targets appearing at unexpected locations [53]. The results of the present study, taken together with the results of previous studies, suggest that the TPJ plays a general role in identifying novel or otherwise salient stimuli in the sensory environment that deviates from expectation.

Activity in the IPL presumably reflects the increased attentional demand caused by a violation of contextual expectations when objects are presented in abnormal contexts. Previous studies have shown that competing attentional subsystems work relative to each other in conflict-trials vs. non-conflict trials [54], [55]. It has been suggested [55] that conflict-trials and selective attention involve distinct neural systems, which is consistent with the present findings.

The pattern of activation is significantly different when objects are presented in their normal contexts. In this case the activity did not involve frontal cortex but instead LOC bilaterally. Although we do not report activity in the LOC for the opposite contrast [abnormal>normal] the LOC was activated when objects were presented in abnormal context in comparison with an object-free baseline. We found that the LOC was significantly less active under abnormal than normal context. Generally, the LOC is a region that is believed to subserve object properties and shape analysis [56]–[58]. The activation of the LOC suggests that this region is not only sensitive to object properties but also to contextual information. Alternatively, the results could imply that elements related to the compositional structure of the original, i.e. normal conditions, may have resulted in a signal increase compared to the altered stimuli (i.e. abnormal conditions). This could be caused by irregularities in low-level visual features in abnormal conditions where a violation of basic image statistics occur more often compared to normal conditions.

Stimuli containing objects in normal contexts also elicited a stronger activation in bilateral parahippocampal gyri. Specifically, we observed a bilateral activation of the parahippocampal place area (PPA) [59], a region of cortex that has been found to respond selectively to houses, landscapes and other environmental sceneries. Our activation occupied a larger number of voxels on the right, which can be explained in light of previous studies showing that the right parahippocampal gyrus is most active during memory for landmarks [60], [61]. It has however been suggested that the PPA, rather than being involved in processing scenes and landmarks specifically, is involved in processing contextual associations more generally [8]–[11]. Indeed, it has been shown that the PPA is sensitive to objects when presented with contextual information, whereas the retrosplenial cortex did not show this sensitivity, since the strength of activity was equivalent regardless of the presence of contextual information in this region [11]. In our study we found activity in the retrosplenial cortex for the contrast [abnormal>normal]. Our results suggest, in accordance with previous studies [11], that there is a bifurcation of contextual information processing, with the PPA being significantly more sensitive to normal visual appearance, and the retrosplenial cortex responding relatively stronger to abnormal contextual processing. This suggest that contextual processing occurs in the retrosplenial cortex regardless of specific contextual properties, although we are unable to confirm this possibility as we did not present control conditions with isolated objects without context. Previous studies demonstrating parahippocampal activation during contextual associations have used behavioural tasks that required subjects to attribute explicit attention to context-related information in the stimuli used [11], [14]. By contrast, in the present study subjects were instructed to rate each stimulus according to aesthetic value, which encourages subjects, although implicitly, to ignore context-related information. Despite these constrictions we observed significant activity in the cortical context network [9] such as the parahippocampal gyrus and the retrosplenial cortex [11], [14] suggesting a robust response to contextual information in this network of areas. Critically, for the context contrasts we controlled for the distribution of aesthetic ratings such that the distribution of ratings were identical for both normal and abnormal conditions. Thus differences in aesthetic rating cannot account for the activations reported.
